# Knowledge, attitudes and practices of Brazilian obstetricians in relation to childbirth care

**DOI:** 10.1002/ijgo.70712

**Published:** 2026-01-07

**Authors:** Rosa Maria Soares Madeira Domingues, Marcos Augusto Bastos Dias, Romina Margarita Hamui, Maria Esther de Albuquerque Vilela, Rodolfo Pacagnela, Ana Paula Esteves‐Pereira, Maria do Carmo Leal

**Affiliations:** ^1^ Instituto Nacional de Infectologia Evandro Chagas Fundação Oswaldo Cruz Rio de Janeiro Brazil; ^2^ Departamento de obstetrícia Instituto Nacional de Saúde da Mulher, da Criança e do Adolescente Fernandes Figueiras/Fiocruz Rio de Janeiro Brazil; ^3^ Instituto de Saúde Coletiva Universidade Federal da Bahia Salvador Brazil; ^4^ Departamento de Ações Nacionais e Cooperação Instituto Nacional de Saúde da Mulher, da Criança e do Adolescente Fernandes Figueiras/Fiocruz Rio de Janeiro Brazil; ^5^ Departamento de Obstetrícia Universidade de Campina Campinas Brazil; ^6^ Departamento de Epidemiologia e Métodos Quantitativos Em Saúde Escola Nacional de Saúde Pública Sergio Arouca/Fiocruz Rio de Janeiro Brazil

**Keywords:** attitudes, Brazil, delivery, health knowledge, obstetric, obstetricians, practice

## Abstract

**Objective:**

To analyze the knowledge, attitudes, and practices of obstetricians regarding childbirth care.

**Methods:**

This was a nationwide cross‐sectional study involving 1267 physicians working in 393 public and private hospitals in Brazil. A self‐administered and anonymous questionnaire, developed specifically for this study, was used. Differences in proportions among obstetricians working in public, mixed, and private services were analyzed using the chi‐square test, with a significance level of 0.05.

**Results:**

High levels of knowledge, favorable attitudes, and adoption of best practices in childbirth care were observed for diet, movement, the presence of a companion, and non‐pharmacological methods for pain relief. Limitations were identified regarding the participation of nurse‐midwives in childbirth care, the use of instrumental delivery, promotion of upright birth positions, and the use of episiotomy. Fear of legal action and unnecessary indications for cesarean sections were frequently reported by professionals as reasons for the country's high cesarean section rates, and should be addressed in medical schools and specialization courses. A more positive perception of cesarean delivery as safe, and the defense of cesarean section as a woman's right, were more common in private services, where there was also less acceptance of births attended by nurse‐midwives.

**Conclusion:**

Differences observed between public and private services may inform the development of new strategies and strengthen ongoing initiatives in Brazil, with the aim of reducing cesarean section rates and promoting best practices in childbirth care.

## 1 INTRODUCTION

Brazil has one of the highest cesarean section rates in the world – 59.6% in 2023 – and cesarean sections have been the predominant mode of delivery since 2009.^1^ Robson groups 2, 5 and 10 contribute most to the overall cesarean rate, demonstrating the importance of preventing the first cesarean section and appropriate management of women with previous cesarean sections and preterm births.^2^

The country has a Unified Health System, which is free of charge and ensures universal access, currently covering about 80% of all births. Private health services complement this system, funded through health insurance or direct payment. The organization of childbirth care differs between the two systems. In public services, pregnant women are attended by health teams working in shifts, who are generally not the same professionals that provided prenatal care. In private services, women are often cared for by the same physician throughout pregnancy and childbirth. These differences can affect the knowledge, attitudes and practices of professionals in both systems, with more favorable attitudes towards cesarean sections in private services, since cesarean section in these services reach 80% of births.^3^

A nationwide study conducted in 2011–2012 revealed a childbirth care model characterized by limited use of best practices, excessive interventions, little participation of nurse‐midwives, and high rates of cesarean sections.^4^, ^5^ Since then, strategies have been implemented in both the public and private sectors to expand best practices^6^, ^7^, ^8^ and reduce unnecessary cesarean sections.^9^, ^10^ Evaluation studies carried out in 2017–2018 found positive results, including increased adoption of best practices and reduced unnecessary interventions and antepartum cesarean sections.^11^

Currently, obstetricians are the main professionals responsible for childbirth care in Brazil. However, there are no previous national studies that have assessed their knowledge, attitudes, and practices (KAP) in the context of these ongoing changes. The aim of this study was to evaluate the knowledge, attitudes, and practices of Brazilian obstetricians regarding childbirth care, according to the type of hospital.

## 2 MATERIALS AND METHODS

This was a prospective cross‐sectional, hospital‐based, nationwide study conducted between November 2021 and March 2025, as part of the “Birth in Brazil II: National Research on Abortion, Labor and Childbirth (NBII)” study.^12^

### 2.1 Study population

Obstetricians providing care to women hospitalized for childbirth. Professionals working exclusively in management or outpatient care were excluded.

### 2.2 Sampling plan

For the NBII survey, a two‐stage probabilistic sample was selected (hospitals and women), with 393 hospitals stratified by the five Brazilian macro‐regions, location (metropolitan or non‐metropolitan), type (public, mixed, private), and size (100–499 or ≥500 births/year), resulting in 60 sampling strata. More information on the NBII sampling plan is available in Leal et al.^12^

All hospitals selected for the NBII were included in this KAP study. We used a convenience sample, due to difficulties in obtaining complete lists of obstetricians from each hospital, particularly in services with open staff models. We included two obstetricians in hospitals with 100–499 births/year and four obstetricians in hospitals with ≥500 births/year.

### 2.3 Data collection

In each hospital, the coordinator of the obstetrics service and first available professionals were invited by the research team. Participation was voluntary, via a self‐administered and anonymous questionnaire, focusing on key issues in the Brazilian context, such as high cesarean section rates, use of best practices, and the limited role of nurse‐midwives (Appendix S1). Professionals who declined, did not return the questionnaire, or returned it blank were considered refusals. Replacements were used for refusals until the sample size was reached in each hospital or when no more professionals were available.

### 2.4 Statistical analysis

Knowledge, attitudes, and practices were analyzed according to hospital type (public = hospitalizations paid for with public funding; private = hospitalizations paid by health insurance or direct payment; mixed = private hospitals with hospitalizations through public and private funding).

We used weighting and calibration procedures. For weighting, because information on the total population of obstetricians involved in childbirth care was unavailable, selection probabilities could not be directly estimated. As a proxy, we used the number of live births, based on the assumption that hospitals with more births would proportionally have more obstetricians. Basic weights were calculated as the inverse of the selection probability, defined as the ratio between the total number of annual live births within each of the 60 strata and the number of sampled obstetricians. Finally, weights were calibrated using data from all hospitals with ≥100 live births in the 2022 Live Birth Information System, based on the joint distribution of region and hospital type. Differences in proportions were assessed using the chi‐square test (α = 0.05), with 95% confidence intervals (CIs) calculated. The proportion of missing values was very low (<3%), and those cases were excluded from the analyses. All analyses accounted for the complex sampling design by using the Complex Samples module of IBM SPSS Statistics, version 26.0 (IBM Corp., Armonk, NY, USA).

### 2.5 Ethical aspects

The NBII study was approved by the National Research Ethics Committee (CONEP), no. 21633519.5.0000.5240, on June 22, 2020. For this study, an invitation letter replaced the standard informed consent form to ensure anonymity. Returning the completed questionnaire was considered agreement to participate. The use of the invitation letter was approved by CONEP.

## 3 RESULTS

A total of 1267 obstetricians were included, representing 86% of the planned sample. Most professionals worked in public hospitals, located mainly in the Southeast and Northeast regions of the country, in hospitals with 500 or more births per year, and in teaching hospitals. The majority of participants were women, with a mean age of 43.3 years, self‐identified as white, married or cohabiting, and with children. Almost 90% (1094/1257) had completed a specialization in obstetrics, and 39.8% (502/1261) had graduated less than 10 years earlier. Most had formal employment contracts, and 85% (1074/1267) worked on shifts. Significant differences were observed by region, hospital size, teaching status, skin color, time since graduation, type of employment contract, and shift work (Table 1).

**TABLE 1:** Characteristics of obstetricians. Brazil, 2021–2025.

Characteristics/type of service	Public (*N* = 575), *n* (%: 95% CI)	Mixed (*N* = 457), *n* (%: 95% CI)	Private (*N* = 235), *n* (%: 95% CI)	Total (*N* = 1267), *n* (%: 95% CI)	*P* value^a^
Region					
North	96 (16.6:14.6–18.8)	28 (6.1:5.3–7.1)	13 (5.6: 4.6–6.9)	137 (10.8:9.7–11.9)	<0.001
Northeast	208 (36.1:34.0–38.3)	95 (20.7:18.6–23.0)	43 (18.4:15.2–22.1)	346 (27.3:25.9–28.7)	
Southeast	181 (31.5:29.5–33.7)	189 (41.4:39.2–43.7)	121 (51.7:47.8–55.5)	492 (38.8:37.4–40.3)	
South	44 (7.6:7.0–8.4)	109 (23.8:21.7–26.1)	29 (12.4:11.1–13.9)	182 (14.4:13.4–15.3)	
Midwest	47 (8.17.2–9.1)	36 (7.9:6.8–9.2)	28 (11.9:8.5–16.5)	111 (8.7:7.8–9.8)	
Hospital size					
100–499 births/year	10 (1.7:1.3–2.3)	6 (1.2:1.0–1.5)	10 (4.2:3.3–5.4)	26 (2.0:1.7–2.3)	<0.001
≥500 births/year	565 (98.3:97.7–98.7)	451 (98.8:98.5–99.0)	225 (95.8:94.6–96.7)	1241 (98.0:97.7–98.3)	
Teaching hospital	437 (77.1:70.5–82.7)	335 (75.2:67.5–81.6)	90 (39.0:29.7–49.3)	862 (69.4:65.0–73.4)	<0.001
Age in years					
Mean (SD)	43.9 (13.2)	42.5 (13.4)	43.15 (12.7)	43.26 (13.2)	<0.398
≤ 35	197 (34.5:29.4–40.1)	178 (39.0:33.6–44.8)	75 (32.3:26.2–39.1)	450 (35.7:32.4–39.2)	
36–54	248 (43.5:38.2–48.9)	189 (41.4:36.5–46.5)	114 (48.9:42.7–55.1)	551 (43.7:40.5–47.0)	
≥55	125 (22.0:18.1–26.5)	89 (19.5:15.5–24.3)	44 (18.8:14.0–24.9)	258 (20.5:18.0–23.3)	
Gender					
Male	239 (41.8:36.9–46.8)	191 (41.7:36.6–47.0)	83 (35.5:29.8–41.7)	513 (40.6:37.5–43.8)	0.288
Female	333 (58.2:53.2–63.1)	266 (58.3:53.0–63.4)	151 (64.5:58.3–70.2)	750 (59.4:56.2–62.5)	
Race/skin color					
White	355 (62.2:57.4–66.8)	324 (70.9:65.5–75.7)	178 (75.9:69.4–81.3)	857 (67.9:64.8–70.8)	0.002
Mixed	192 (33.6:29.3–38.2)	115 (25.1:20.6–30.3)	46 (19.7:15.0–25.4)	353 (27.9:25.2–30.9)	
Black	16 (2.8:1.6–4.7)	12 (2.7:1.5–4.9)	4 (1.6:0.6–4.3)	32 (2.5:1.8–3.7)	
Asian	8 (1.4:0.7–2.8)	4 (0.8:0.3–2.5)	7 (2.8:1.2–6.8)	19 (1.5:0.9–2.4)	
Indigenous	0	2 (0.5:0.1–2.1)	0	2 (0.2:0.0–0.8)	
Conjugal situation					
Married/cohabiting	395 (68.9:64.3–73.1)	316 (69.1:63.6–74.1)	175 (74.3:68.2–79.6)	886 (70.:66.9–72.80)	0.361
Single/widowed/divorced	179 (31.1:26.9–35.7)	141 (30.9:25.9–36.4)	60 (25.7:20.4–31.8)	380 (30.0:27.2–33.1)	
Have children	374 (65.8:60.7–70.6)	278 (61.1:55.3–66.7)	148 (63.6:56.6–70.1)	800 (63.7:60.4–67.0)	0.436
Time since graduation (years)					
<10	225 (39.4:33.9–45.1)	198 (43.5:37.3–49.9)	79 (33.6:27.3–40.6)	502 (39.8:36.2–43.5)	0.004
10–19	108 (18.9:15.4–23.0)	104 (22.9:18.7–27.7)	77 (32.8:27.6–38.6)	289 (22.9:20.5–25.6)	
>20	238 (41.7:36.7–46.8)	153 (33.6:28.2–39.4)	79 (33.5:26.6–41.3)	470 (37.3:34.0–40.7)	
Specialization in obstetrics	497 (86.9:83.2–89.8)	387 (85.4:80.8–89.0)	210 (90.6:86.5–93.5)	1.094 (87.0:84.7–89.1)	0.221
Employment contract					
Medical residency	38 (6.6:4.3–10.0)	48 (10.5:7.0–15.3)	5 (2.2:0.9–5.2)	91 (7.2:5.4–9.4)	<0.001
Public servant	143 (25.1:20.0–31.0)	10 (2.2:0.8–5.8)	5 (2.2:0.9–5.1)	158 (12.6:10.1–15.5)	
Autonomous	25 (4.3:2.7–6.8)	53 (11.6:7.9–16.6)	48 (20.7:15.6–26.9)	126 (10.0:8.1–12.3)	
Contract	362 (63.3:56.9–69.2)	339 (74.5:68.5–79.7)	164 (70.6:63.8–76.6)	865 (68.7:64.9–72.2)	
Others	4 (0.7:0.2–2.1)	6 (1.3:0.4–4.2)	10 (4.3:2.4–7.7)	20 (1.6:1.0–2.6)	
Work in shifts	488 (84.8:81.4–87.7)	405 (88.6:83.7–92.2)	181 (77.0:70.0–82.9)	1074 (84.8:82.2–87.0)	0.007
Work daily	88 (15.3:11.9–19.3)	64 (13.9:9.9–19.3)	41 (17.3:12.3–23.9)	193 (15.2:12.8–18.0)	0.660

Abbreviations: CI, confidence interval; SD, standard deviation.

^a^ Chi‐square test.

Almost all obstetricians reported having received training in induction of labor with misoprostol and oxytocin, with lower rates for mechanical induction (86%, 1037/1205), instrumental delivery (82.9%, 1033/1246), and childbirth care in upright positions (73.3%, 900/1228), with significant differences across services. Approximately 60% reported training in best practices for childbirth care within their health service. Knowledge of the Brazilian Ministry of Health (MoH) and WHO childbirth care guidelines was low. Nearly all professionals demonstrated adequate knowledge about diet, episiotomy, and respecting the woman's chosen position during labor and birth (Table 2).

**TABLE 2:** Knowledge of obstetricians in childbirth care. Brazil, 2021–2025.

Knowledge/type of hospital	Public (*N* = 575), *n* (%: 95% CI)	Mixed (*N* = 457), *n* (%: 95% CI)	Private (*N* = 235), *n* (%: 95% CI)	Total (*N* = 1267), *n* (%: 95% CI)	*P* value^a^
Medical training					
Induction of labor with misoprostol	518 (91.8:88.8–94.1)	432 (95.1:91.8–97.1)	222 (96.8:93.4–98.5)	1172 (94.0:92.2–95.3)	0.039
Induction of labor with oxytocin	567 (99.5:98.5–99.8)	452 (99.4:98.0–99.8)	229 (99.3:97.0–99.8)	1248 (99.4:98.8–99.7)	0.935
Induction of labor with mechanical methods	466 (85.8:82.5–88.6)	373 (84.4:79.1–88.5)	198 (89.5:85.3–92.6)	1037 (86.0:83.5–88.1)	0.265
Instrumental delivery	451 (79.6:74.9–83.7)	381 (85.0:80.5–88.5)	201 (87.3:81.6–91.3)	1033 (82.9:80.2–85.4)	0.047
Childbirth care in upright positions	387 (69.4:64.5–73.9)	337 (76.3:71.4–80.6)	176 (76.8:71.5–81.4)	900 (73.3:70.3–76.0)	0.038
In‐service training					
Best practices in childbirth care	331 (61.1:55.8–66.2)	226 (54.5:48.0–60.8)	142 (62.5:54.3–70.1)	699 (59.1:55.4–62.7)	0.183
Knowledge of clinical guideline					
Brazilian MoH vaginal delivery guideline					
Don't know/never read	74 (12.9:10.3–16.0)	36 (8.0:5.7–11.2)	40 (17.1:13.1–22.1)	150 (11.9:10.2–13.9)	0.017
Read partially	278 (48.5:43.5–53.4)	213 (46.7:41.5–52.0)	105 (45.0:37.6–52.6)	596 (47.2:44.0–50.5)	
Read completely	222 (38.7:33.6–44.0)	206 (45.2:40.2–50.4)	88 (37.9:31.0–45.3)	516 (40.9:37.7–44.2)	
Brazilian MoH cesarean section guideline					
Don't know/never read	124 (21.7:18.6–25.2)	81 (17.9:14.1–22.6)	61 (25.9:21.1–31.3)	266 (21.1:18.9–23.6)	0.260
Read partially	237 (41.4:36.7–46.3)	197 (43.3:38.0–48.8)	87 (37.3:31.3–43.6)	521 (41.3:38.2–44.5)	
Read completely	211 (36.9:31.9–42.1)	176 (38.8:33.6–44.1)	86 (36.9:30.4–43.9)	473 (37.6:34.4–40.8)	
WHO intrapartum care guideline					
Don't know/never read	240 (42.2:37.4–47.1)	171 (37.8:32.7–43.1)	82 (35.2:29.7–41.2)	493 (39.3:36.3–42.4)	0.455
Read partially	191 (33.5:29.6–37.6)	167 (37.0:31.5–42.7)	85 (36.4:30.8–42.5)	443 (35.3:32.4–38.3)	
Read completely	138 (24.3:20.0–29.3)	115 (25.3:20.9–30.3)	66 (28.4:22.6–34.9)	319 (25.4:22.6–28.5)	
Adequate knowledge about best practices					
Oral fluid and food intake during labor	539 (96.0:93.8–97.4)	433 (96.3:93.7–97.8)	222 (96.4:93.8–98.0)	1194 (96.2:94.9–97.1)	0.938
Restricted use of episiotomy	564 (98.9:97.2–99.5)	445 (98.1:95.6–99.2)	227 (98.9:96.9–99.6)	1236 (98.6:97.6–99.2)	0.597
Respect for the chosen position during labor and childbirth	547 (97.2:95.5–98.3)	440 (99.0:97.6–99.6)	227 (98.6:94.9–99.6)	1214 (98.1:97.2–98.7)	0.155

Abbreviations: CI, confidence interval; MoH, Ministry of Health.

^a^ Chi‐square test.

Around 80% agreed that nurse‐midwives can provide childbirth care (1013/1257) and that vaginal delivery (1068/1258) is the safest option for low‐risk women. Nearly half of the participants (677/1260) supported the right of women to request a cesarean section, believed lawsuits were more common after vaginal births (695/1257), and reported performing a cesarean section instead of a vaginal or instrumental delivery to avoid litigation (698/1258). The most frequently cited reasons for the increase in cesarean sections in Brazil were: “cesarean sections on request,” “fear of lawsuits,” “convenience of the obstetrician” and “unnecessary indications.” The main reasons given for cesarean sections on request were fear of pain, fear of vaginal birth, a previous negative vaginal birth experience, and convenience for the woman. Obstetricians in private services reported lower agreement with nurse‐midwife–led childbirth care; were more likely to perceive vaginal and cesarean deliveries as equally safe; more strongly supported women's right to choose a cesarean section; and more often cited “low remuneration for vaginal delivery” and “cesarean sections on request” as reasons for the rising cesarean section rate. A higher proportion cited “convenience for the woman,” “having the doctor of choice” and “preservation of the perineum” in private and mixed hospitals, while “tubal ligation” was more often cited in public and mixed hospitals (Table 3).

**TABLE 3:** Attitudes of obstetricians regarding aspects of labor and chilbirth care. Brazil, 2021–2025.

Attitudes/type of hospital	Public (*N* = 575), *n* (%: 95% CI)	Mixed (*N* = 457), *n* (%: 95% CI)	Private (*N* = 235), *n* (%: 95% CI)	Total (*N* = 1267), *n* (%: 95% CI)	*P* value^a^
Childbirth care by nurse‐midwives					
Agree	469 (82.5:77.8–86.4)	374 (82.3:77.2–86.4)	170 (72.5:66.5–77.8)	1013 (80.6:77.6–83.2)	0.016
Disagree	71 (12.4:9.1–16.7)	54 (11.9:8.4–16.5)	45 (19.4:15.2–24.4)	170 (13.5:11.3–16.1)	
No opinion	29 (5.1:3.5–7.3)	27 (5.8:3.7–9.1)	19 (8.1:5.2–12.6)	75 (5.9:4.6–7.5)	
Safest type of delivery for low risk pregnant women					
Vaginal	505 (88.5:85.3–91.1)	380 (83.7:79.1–87.4)	183 (78.4:72.6–83.3)	1068 (84.9:82.5–87.0)	0.006
Cesarean	7 (1.2:0.5–2.9)	12 (2.6:1.3–4.9)	5 (2.0:0.9–4.6)	24 (1.9:1.2–2.9)	
Both are equally safe	48 (8.5:6.2–11.5)	59 (13.1:10.0–17.0)	42 (17.8:13.3–23.4)	149 (11.9:10.0–14.0)	
No opinion	10 (1.8:1.0–3.2)	3 (0.6:0.2–2.0)	4 (1.8:0.7–4.4)	17 (1.4:0.9–2.2)	
Women should have the right for a cesarean section on request					
Agree	271 (47.4:42.3–52.6)	228 (50.1:44.2–56.0)	178 (76.2:69.9–81.5)	677 (53.7:50.3–57.1)	<0.001
Disagree	243 (42.6:37.5–47.8)	181 (39.8:34.1–45.8)	46 (19.4:14.5–25.4)	470 (37.3:34.0–40.7)	
No opinion	57 (10.0:7.7–13.0)	46 (10.1:7.1–14.2)	11 (4.4:2.4–8.1)	114 (9.0:7.3–11.0)	
Lawsuits are more common in					
Vaginal births	305 (53.6:49.0–58.1)	260 (57.2:51.8–62.5)	130 (55.7:48.6–62.5)	695 (55.3:52.1–58.4)	0.334
Cesarean section	22 (3.8:2.3–6.1)	18 (4.0:2.1–7.4)	6 (2.6:1.2–5.8)	46 (3.6:2.6–5.2)	
Both	204 (35.9:31.4–40.7)	162 (35.6:30.6–41.0)	88 (37.8:30.8–45.2)	454 (36.1:33.1–39.3)	
No opinion	38 (6.7:4.6–9.7)	15 (3.2:2.0–5.3)	9 (4.0:2.3–6.7)	62 (4.9:3.8–6.5)	
Has ever indicated a cesarean section instead of vaginal delivery to avoid legal action					
Yes	326 (57.2:52.3–62.0)	261 (57.4:51.7–63.0)	111 (47.4:40.5–54.4)	698 (55.5:52.1–58.7)	0.146
No	219 (38.6:34.0–43.4)	174 (38.2:32.7–44.0)	114 (48.6:42.1–55.2)	507 (40.3:37.1–43.5)	
Not informed	24 (4.2:2.8–6.3)	20 (4.4:2.6–7.5)	9 (4.0:2.3–6.8)	53 (4.2:3.2–5.6)	
Has ever indicated a cesarean section instead of instrumental delivery to avoid legal action					
Yes	265 (46.7:41.7–51.7)	228 (50.3:44.5–56.1)	98 (42.2:35.0–49.7)	591 (47.2:43.8–50.6)	0.023
No	238 (41.9:36.9–47.1)	172 (37.9:32.4–43.6)	122 (52.4:44.7–60.0)	532 (42.4:39.0–45.8)	
Never performed an instrumental delivery	65 (11.4:8.4–15.3)	54 (11.8:8.4–16.5)	12 (5.4:3.0–9.4)	131 (10.4:8.4–12.9)	
Reasons for the increase of cesarean sections rates in Brazil					
Cesarean on request	430 (74.7:70.5–78.5)	366 (80.1:70.6–83.7)	197 (83.7:79.0–87.5)	993 (78.3:75.8–80.8)	0.011
Fear of lawsuits	344 (59.9:55.1–64.5)	272 (59.5:54.8–64.1)	129 (54.8:48.6–61.0)	745 (58.8:55.8–61.7)	0.427
Convenience of the obstetrician	312 (54.2:49.2–59.1)	251 (54.8:49.6–60.0)	145 (61.9:54.9–68.3)	708 (55.9:52.6–59.0)	0.195
Unnecessary indications	277 (48.2:43.0–53.5)	242 (52.9:46.9–58.8)	110 (46.7:40.2–53.4)	629 (49.6:46.2–53.1)	0.326
Lack of adequate structure/team	215 (37.4:33.3–41.8)	173 (37.8:32.6–43.3)	80 (34.1:26.9–42.2)	468 (36.9:33.9–40.1)	0.700
Low remuneration for vaginal delivery	182 (31.7:27.0–36.9)	130 (28.3:24.0–33.1)	98 (41.6:34.6–49.0)	410 (32.3:29.3–35.5)	0.011
Increased safety of cesarean section	105 (18.3:14.6–22.6)	83 (18.1:15.0–21.7)	46 (19.5:14.1–26.2)	234 (18.4:16.1–21.0)	0.921
Urban violence	71 (12.4:9.4–16.2)	44 (9.6:6.6–13.8)	30 (12.7:9.4–16.9)	145 (11.4:9.5–13.7)	0.389
Inadequate prenatal care	31 (5.3:3.6–7.9)	20 (4.4:2.6–7.2)	10 (4.2:2.3–7.7)	61 (4.8:3.6–6.3)	0.735
Pro‐cesarean culture	6 (1.0:0.4–2.6)	2 (0.5:0.1–2.0)	2 (1.0:0.3–4.2)	11 (0.8:0.4–1.7)	0.657
Reasons for cesarean section on request					
Fear of pain	502 (87.2:83.7–90.1)	402 (88.1:84.6–90.8)	204 (86.7:82.1–90.3)	1108 (87.4:85.3–89.3)	0.867
Fear of vaginal delivery	447 (77.6:73.7–81.1)	361 (79.0:74.8–82.7)	189 (80.5:74.4–85.4)	997 (78.7:76.1–81.0)	0.674
Previous negative experience with VD	365 (63.5:59.1–67.8)	296 (64.8:59.0–70.2)	148 (62.9:56.1–69.3)	809 (63.9:60.7–66.9)	0.890
Convenience for the woman	265 (46.1:41.4–50.9)	223 (48.8:43.7–53.9)	150 (63.6:56.4–70.2)	638 (50.3:47.2–53.4)	<0.001
Tubal ligation	303 (52.7:47.8–57.5)	238 (52.0:46.5–57.3)	79 (33.7:27.2–40.8)	620 (48.9:45.7–52.1)	<0.001
Having the doctor of choice	191 (33.2:28.5–38.2)	177 (38.8:33.5–44.4)	107 (45.4:38.3–52.8)	475 (37.5:34.3–40.8)	0.020
Previous positive experience with cesarean	186 (32.3:27.9–37.1)	162 (35.4:31.0–40.0)	96 (40.8:33.9–48.2)	444 (35.0:32.1–38.0)	0.121
Preserve perineum	114 (19.9:16.4–24.0)	124 (27.1:22.4–32.3)	62 (26.3:20.1–33.5)	300 (23.7:21.0–26.6)	0.053
Baby safety	66 (11.4:8.6–15.1)	62 (13.5:10.2–17.5)	22 (9.5:6.8–13.2)	150 (11.8:9.9)	0.335
Women's safety	30 (5.2:3.3–8.1)	35 (7.7:5.4–10.9)	9 (3.8:2.2–6.7)	74 (5.8:4.5–7.5)	0.120
Secure hospital bed	14 (2.4:1.3–4.3)	12 (2.7:1.5–4.8)	10 (4.3:2.4–7.6)	36 (2.9:2.0–4.0)	0.366

Abbreviations: CI, confidence interval; VD, vaginal delivery.

^a^ Chi‐square test.

Most obstetricians worked in hospitals with childbirth care guidelines, though only 44.1% (380/862) reported their routine use. Fewer than half (594/1267) worked in services where nurse‐midwives were always available, and one‐quarter (242/970) did not collaborate with them. Approximately 90% of professionals reported adopting several best practices, though with lower adherence for early amniotomy (67.7% [849/1254] “never”) and upright positions during childbirth (55.1% [690/1252] “always”). Professionals in private services reported greater routine use of guidelines; greater use of “presence of a companion” and “upright positions”; less use of episiotomy; lower availability of nurse‐midwives; and less collaborative practice (Table 4).

**TABLE 4:** Obstetricians' practices in labor and childbirth care. Brazil, 2021–2025.

Practices/type of hospital	Public (*N* = 575), *n* (% 95% CI)	Mixed (*N* = 457), *n* (% 95% CI)	Private (*N* = 235), *n* (% 95% CI)	Total (*N* = 1267), *n* (% 95% CI)	*P* value^a^
Availability of childbirth care guideline					
Yes	381 (66.3:60.5–71.7)	313 (68.4:62.4–73.8)	168 (71.4:63.3–78.3)	862 (68.0:64.3–71.4)	0.575
No/don't know	194 (33.7:28.3–39.5)	144 (31.6:26.2–37.6)	67 (28.6:21.7–36.7)	405 (32.0:28.6–35.7)	
Team use of childbirth care guideline^b^					
Always	163 (42.8:36.8–49.0)	123 (39.3:32.4–46.7)	94 (55.8:47.1–64.2)	380 (44.1:40.0–48.2)	0.034
Occasionally	208 (54.4:48.3–60.4)	179 (57.1:49.9–64.1)	68 (40.5:33.2–48.3)	454 (52.7:48.7–56.7)	
Never/don't know	10 (2.8:1.5–5.1)	11 (3.6;1.8–6.9)	6 (3.7:1.7–7.7)	27 (3.2:2.2–4.8)	
Availability of nurse‐midewives for childbirth care					
Always	289 (50.2:43.1–57.3)	211 (46.2:39.6–52.9)	94 (39.9:32.4–48.0)	594 (46.9:42.6–51.2)	0.046
Occasionally	165 (28.7:23.8–34.1)	153 (33.5:27.9–39.6)	63 (26.8:21.4–32.9)	381 (30.1:26.9–33.5)	
Never/don't know	121 (21.1:16.3–26.8)	93 (20.3:14.8–27.2)	78 (33.3:25,8–41.7)	292 (23.1:19.7–26.8)	
Collaborative work with nurse‐midwives^c^					
No	72 (15.9:12.0–20.8)	96 (26.5:21.6–32.1)	74 (47.5:39.1–56.0)	242 (24.9:21.8–28.3)	<0.001
Yes, working together	115 (25.3:20.4–31.0)	129 (35.6:29.7–41.9)	43 (27.5:20.8–35.3)	286 (29.5:26.1–33.2)	
Yes, available on demand	266 (58.7:51.8–65.3)	137 (37.9:31.5–44.6)	39 (25.1:18.5–33.0)	442 (45.5:41.3–49.8)	
Practices in childbirth care					
Freedom of movement					
Always	537 (94.4:92.1–96.1)	428 (94.7:91.7–96.7)	219 (94.1:89.0–96.9)	1184 (94.5:92.9–95.8)	0.987
Occasionally	30 (5.2:3.6–7.5)	23 (5.1:3.2–8.1)	13 (5.6:2.8–10.7)	66 (5.3:4.0–6.9)	
Never	2 (0.3:0.1–1.3)	1 (0.2:0.0–1.2)	1 (0.3:0.0–2.1)	4 (0.3:0.1–0.7)	
Oral fluid and food					
Always	500 (88.0:84.3–90.9)	387 (85.7:81.1–89.3)	205 (88.2:82.3–92.3)	1092 (87.2:84.7–89.3)	0.566
Occasionally	56 (9.8:7.3–13.1)	57 (12.7:9.3–17.2)	25 (10.7;6.7–16.6)	138 (11.0:9.1–13.3)	
Never	13 (2.2:1.2–4.1)	7 (1.6:0.8–3.1)	3 (1.1:0.3–3.3)	23 (1.8:1.2–2.7)	
Non‐pharmacological methods for pain relief					
Always	497 (87.3:83.5–90.3)	413 (91.4:87.7–94.0)	213 (91.5:86.2–94.8)	1123 (89.5:87.3–91.4)	0.198
Occasionally	64 (11.1:8.4–14.6)	36 (8.0:5.5–11.5)	16 (7.0:4.2–11.5)	116 (9.2:7.5–11.3)	
Never	9 (1.6:0.8–3.2)	3 (0.7:0.2–1.9)	4 (1.5:0.6–3.6)	16 (1.2:0.8–2.1)	
Presence of a companion					
Always	506 (88.8:84.9–91.8)	424 (93.7:90.4–95.8)	227 (97.6:94.5–99.0)	1157 (92.2:90.1–93.9)	0.001
Occasionally	55 (9.7:7.0–13.2)	28 (6.1:4.0–9.1)	5 (2.1:0.8–5.2)	88 (7.0:5.5–8.9)	
Never	9 (1.5:0.7–3.2)	1 (0.3:0.0–1.6)	1 (0.3:0.0–1.8)	11 (0.8:0.4–1.6)	
Early amniotomy					
Always	5 (0.9:0.4–2.2)	6 (1.2:0.5–3.2)	3 (1.4:0.5–3.8)	14 (1.1:0.6–1.9)	0.257
Occasionally	192 (33.7:28.6–39.2)	121 (26.7:21.9–32.2)	78 (33.6:27.1–40.8)	391 (31.2:28.0–34.6)	
Never	372 (65.4:60.0–70.5)	326 (72.0:66.7–76.8)	151 (64.9:57.9–71.3)	849 (67.7:64.4–70.9)	
Upright positions during childbirth care					
Always	295 (52.0:46.7–57.3)	243 (53.7:48.2–59.2)	152 (65.1:58.3–71.4)	690 (55.1:51.7–58.4)	0.023
Occasionally	236 (41.6:36.6–46.8)	190 (42.0:36.7–47.4)	75 (32.2:26.5–38.4)	501 (40.0:36.8–43.3)	
Never	36 (6.3:4.5–9.0)	20 (4.3:2.6–7.1)	6 (2.7:1.2–6.1)	62 (4.9:3.8–6.5)	
Episiotomy					
Always	5 (0.9:0.3–2.3)	7 (1.5:0.7–3.4)	1 (0.4:0.1–1.9)	13 (1.0:0.6–1.8)	<0.001
Occasionally	424 (74.8:69.8–79.2)	311 (69.6:63.4–75.1)	127 (54.3:45.9–62.5)	862 (69.1:65.6–72.4)	
Never	138 (24.3:19.9–29.3)	129 (28.9:23.2–35.3)	106 (45.2:37.1–53.6)	373 (29.9:26.5–33.4)	

Abbreviation: CI, confidence interval.

^a^ Chi‐square test.

^b^ When guideline is available (*N* = 861, public = 381, mixed = 313, private = 168).

^c^ When nurse‐midwives are available for childbirth care (*N* = 975, public = 454, mixed = 364, private = 157).

The main facilitators of best childbirth care were commitment from professionals and managers, availability of a multidisciplinary team, supportive hospital environment, existence of guidelines, and adequate supplies and equipment—all more frequently reported in private services. The most frequently cited barriers were staff shortages, high hospital admission volumes, and lack of supplies and equipment, with higher proportions in public hospitals (Table 5).

**TABLE 5:** Facilitators and barriers to labor and childbirth care by hospital type. Brazil, 2021–2025.

Items/type of hospital	Public (*N* = 575), *n* (% 95% CI)	Mixed (*N* = 457), *n* (% 95% CI)	Private (*N* = 235), *n* (% 95% CI)	Total (*N* = 1267), *n* (% 95% CI)	*P* value^a^
Facilitators					
Commitment of professionals	413 (71.8:67.4–75.8)	307 (67.2:61.7–72.2)	175 (74.6:68.5–79.9)	895 (70.6:67.7–73.5)	0.148
Availability of a multidisciplinary team	297 (51.6:45.5–57.7)	261 (57.2:51.6–62.6)	154 (65.3:58.1–71.9)	712 (56.2:52.5–59.8)	0.019
Commitment of service managers	271 (47.1:41.3–53.1)	190 (41.6:36.1–47.4)	133 (56.6:49.3–63.7)	594 (46.9:43.3–50.6)	0.013
Supportive hospital environment	202 (35.1:30.2–40.4)	206 (45.0:39.6–50.6)	125 (53.2:46.4–60.0)	533 (42.1:38.8–45.4)	<0.001
Supplies	161 (28.0:23.2–33.3)	156 (34.1:29.1–39.4)	147 (62.6:54.9–69.7)	464 (36.6:33.3–40.0)	<0.001
Clinical guidelines	194 (33.7:28.2–39.5)	153 (33.5:27.7–39.7)	103 (43.9:36.2–51.9)	450 (35.5:31.9–39.2)	0.012
Equipment/furniture	123 (21.4:17.0–26.6)	148 (32.5:27.5–37.8)	129 (54.7:47.2–62.0)	400 (31.6:28.4–34.9)	<0.001
Management strategies	120 (20.8:17.1–25.0)	93 (20.3:16.1–25.3)	66 (28.3:22.7–34.8)	279 (22.0:19.5–24.8)	0.081
Number of professionals	118 (20.5:16.5–25.2)	88 (19.2:15.2–24.0)	62 (26.3:20.7–32.7)	268 (21.1:18.5–24.0)	0.180
Number of hospital admissions	64 (11.2:8.4–14.7)	73 (15.9:12.5–20.1)	36 (15.2:11.5–19.7)	173 (13.6:11.7–15.9)	0.093
Others	28 (4.9:3.2–7.5)	19 (4.2:2.5–6.9)	8 (3.2:1.5–6.9)	55 (4.4:3.2–5.9)	0.619
Barriers					
Number of professionals	257 (44.7:39.4–50.1)	189 (41.4:36.1–46.9)	84 (35.5:29.2–42.4)	530 (41.8:38.5–45.2)	0.128
Number of hospital admissions	270 (47.0:41.2–53.0)	163 (35.6:29.6–42.2)	53 (22.4:16.7–29.3)	486 (38.4:34.7–42.1)	<0.001
Supplies	269 (46.7:40.9–52.6)	148 (32.4:26.7–38.8)	24 (10.1:6.9–14.5)	441 (34.8:31.3–38.4)	<0.001
Equipment/furniture	247 (43.0:37.6–48.5)	124 (27.1:22.1–32.7)	22 (9.4:6.1–14.1)	393 (31.0:27.8–34.4)	<0.001
Clinical guidelines	147 (25.6:21.5–30.2)	102 (22.3:18.2–27.0)	45 (19.3:14.0–25.8)	294 (23.2:20.6–26.1)	0.228
Management strategies	128 (22.2:18.3–26.7)	112 (24.5:20.1–29.5)	47 (20.0:14.9–26.2)	287 (22.6:20.0–25.5)	0.501
Commitment of service managers	154 (26.7:22.2–31.6)	89 (19.5:15.6–24.2)	37 (15.9:11.2–22.0)	280 (22.1:19.4–25.0)	0.008
Supportive hospital environment	144 (25.1:20.8–29.9)	74 (16.2:12.3–21.0)	18 (7.8:4.9–12.2)	236 (18.7:16.1–21.5)	<0.001
Availability of a multidisciplinary team	118 (20.6:16.8–25.1)	85 (18.6:14.2–23.9)	30 (12.6:8.7–17.9)	233 (18.3:15.8–21.3)	0.091
Commitment of professionals	92 (16.0:12.8–19.9)	68 (14.8:11.9–18.4)	37 (15.9:11.1–22.1)	197 (15.6:13.5–17.9)	0.887
Others	28 (4.9:3.2–7.5)	26 (5.7:3.6–8.8)	27 (11.7:7.7–17.3)	82 (6.4:5.0–8.2)	0.007

Abbreviation: CI, confidence interval.

^a^ Chi‐quare test.

## 4 DISCUSSION

High levels of knowledge, positive attitudes, and the use of best practices in childbirth care were observed, but participation of nurse‐midwives and the use of upright positions remained low. Significant differences were found between services, mainly related to childbirth care provided by nurse‐midwives and perceptions regarding cesarean sections. Notably, key facilitators in private services were often cited as barriers in public services, reflecting structural differences between the two systems.

Clinical guidelines based on scientific evidence should guide practice, and their limited implementation is a barrier to the adoption of best practices.^13^ In this study, obstetricians demonstrated limited knowledge of the Brazilian MoH and WHO guidelines. However, two‐thirds reported the existence of service‐level guidelines, with almost all stating they used them routinely or occasionally. In private services in particular, the existence of guidelines was considered a facilitator of childbirth care. One possible explanation is the implementation of a quality improvement program in Brazilian private hospitals since 2015.^10^ After the first year of this program, the probability of vaginal delivery increased by 37.7%, largely due to reorganization of the childbirth care model and greater access to best practices supported by clinical guidelines.^14^

Obstetricians reported high knowledge, positive attitudes, and frequent use of best practices, particularly regarding diet, freedom of movement, the presence of a companion, and non‐pharmacological methods for pain relief. Previous studies conducted among women in public and private hospitals in 2017^11^ also showed increased use of such practices compared to the 2011/2012 national study, though not reaching the levels observed here. The higher prevalence of companions during childbirth in private services aligns with earlier findings and likely reflects the physical infrastructure of these hospitals.

Encouraging women to choose the most comfortable position during labor and birth, including upright positions, is a WHO recommendation.^6^ However, birth in upright position was the practice with the lowest uptake and the least training during medical school. Thus, although professionals acknowledge that the woman's chosen position should be respected, availability remains limited. This is consistent with national data from 2017 showing that 62% of vaginal births in public services and 70% in private hospitals still occur in the lithotomy position.^11^

Only 1% of obstetricians reported routinely performing episiotomy, consistent with evidence discouraging liberal use.^15^ However, 69% reported using it occasionally, a frequency similar to findings from a previous national study with 1113 obstetricians.^16^ Although episiotomy use has declined in Brazil, 30% of vaginal births in public hospitals and 40% in private hospitals in 2017/2018 still involved the procedure.^11^ Reported barriers to reducing episiotomy include insufficient knowledge and inadequate training to prevent severe perineal tears.^17^

Childbirth care by nurse‐midwives is associated with positive outcomes.^18^ Yet, in Brazil, only 20% of hospital vaginal deliveries between 2012 and 2024 were attended by nurse‐midwives.^1^ In this study, obstetricians working in private services reported less availability of nurse‐midwives, less agreement with their participation in childbirth care, and less collaborative work. These findings are consistent with previous studies conducted among women in 2011/2012 and in 2017 showing higher proportions of nurse‐midwife–assisted births in the public sector, with fewer than 5% in private hospitals.^5^, ^11^ A qualitative study in private hospitals identified several barriers to expanding obstetric nursing, including women's limited awareness of the role of nurse‐midwives, insufficient numbers and training of professionals, and financing issues.^19^

Across all service types, obstetricians cited cesarean sections on request as the main reason for rising cesarean section rates. However, this is inconsistent with both international and national studies, which indicate that most women prefer vaginal birth.^3^, ^20^, ^21^ The reasons attributed by physicians to women's preference—fear of pain, fear of childbirth, and negative past experiences—are supported by national and international literature, reinforcing the importance of prenatal counseling and the use of best practices to ensure a positive birth experience.^3^, ^6^, ^20^, ^21^ Wider access to epidural analgesia, used in fewer than 4% of vaginal births globally and available to fewer than 20% of women in Brazilian public hospitals, could help reduce cesarean sections on request.^11^, ^22^ Requests for cesarean section to enable tubal ligation were more common in public hospitals, consistent with earlier studies, and reflect limited access to reproductive planning services.^3^ Expanding access to reproductive health care, including postpartum vaginal tubal ligation, could help reduce unnecessary cesarean sections.

The other frequently cited reasons – fear of litigation, physician convenience, and unnecessary indications – reflect structural aspects of obstetric training and practice. Fear of lawsuits, reported by about half of respondents, aligns with previous evidence identifying obstetrics as one of the medical specialties with the greatest litigation risk and cost. Cesarean delivery is often adopted as a defensive rather than a clinical measure.^23^, ^24^ A study conducted with 403 obstetricians in Brazil showed that the perception of a higher risk of lawsuits against obstetricians influenced medical decision making and led to a more than six‐fold increase in cesarean sections.^23^ Litigation‐related fear results from complex cognitive, social, and environmental interactions. Cognitive biases – both rational and irrational beliefs about safety and risk – can override professional training, guidelines, and statistical evidence, influencing emotional responses and decisions about the mode of delivery.^24^

### 4.1 Strengths and limitations

The study's strengths include its originality, as the first nationwide investigation of obstetricians' knowledge, attitudes, and practices regarding childbirth care, and the use of a self‐administered, anonymous questionnaire designed to minimize socially desirable responses. Analysis by service type allowed identification of differences that may guide tailored strategies, strengthening ongoing initiatives in the country.

The main limitation is the use of a convenience sample. Although participants' characteristics were consistent with national medical demographics, the sample overrepresented formally employed obstetricians,^25^ which may limit generalizability—especially for private services, where many physicians work without formal contracts and may be less bound by institutional protocols.

## 5 CONCLUSION

Obstetricians reported increased knowledge and adoption of best practices in childbirth care. However, Brazil's persistently high cesarean section rates suggest a practice misaligned with scientific evidence. A more favorable perception of cesarean section and its defense as a woman's right were observed in private services, consistent with the high cesarean section rates in these services. Medical education and specialist training should target litigation, physician convenience and unnecessary indications of cesarean section as these were frequently cited reasons for the high cesarean section rate in the country. Incentives for team‐based care are necessary to increase nurse‐midwives participation in childbirth care.

## AUTHOR CONTRIBUTIONS

Rosa Maria Soares Madeira Domingues: Made substantial contributions to the conception and design of the work, draft the first version and approved the final version. Marcos Augusto Bastos Dias, Romina Margarita Hamui, Maria Esther de Albuquerque Vilela and Rodolfo Pacagnela: Made substantial contribution to the design of the work and interpretation of data; reviewed the work critically and approved the final version. Ana Paula Esteves‐Pereira: Made substantial contribution to the acquisition and analysis of the data, reviewed the work critically and approved the final version. Maria do Carmo Leal: Made substantial contributions to the conception of the work, reviewed the work critically and approved the final version. All authors agreed to be accountable for all aspects of the work in ensuring that questions related to the accuracy or integrity of any part of the work were appropriately investigated and resolved.

## FUNDING INFORMATION

Secretaria de Ciência, Tecnologia e Inovação e do Complexo Econômico‐Industrial da Saúde – SECTICS/MS, TED 145/23, process no. 25000.174797/2023‐14, Conselho Nacional de Desenvolvimento Científico e Tecnológico (CNPq), process no. 443766/2018‐5, FAPERJ—Fundação Carlos Chagas Filho de Amparo à Pesquisa do Estado do Rio de Janeiro, Programa Jovem Cientista do Nosso Estado, process no. E‐26/201.364/2021.

## CONFLICT OF INTEREST STATEMENT

The authors have no conflicts of interest.

## Supporting information


Appendix S1

## Data Availability

Research data are not shared.
