# Supplementary material for: Knowledge, attitudes and practices of Brazilian obstetricians in relation to childbirth care
Source: Int J Gynaecol Obstet. 2026 Jan 7;173(3):1352–61. doi: 10.1002/ijgo.70712 (PMC13173629; doi:10.1002/ijgo.70712)
Supplement: Supplementary file 1 — Appendix S1 [file IJGO-173-1352-s001.pdf]

BIRTH IN BRAZIL II: NATIONAL SURVEY ON *ABORTION*, CHILDBIRTH, AND *BIRTH*

MEDICAL PROFESSIONALS' QUESTIONNAIRE

This questionnaire should be answered anonymously.
You should not write any personal information that could identify you. Please answer honestly.

Questionnaire number *(to be filled in by the research team)*

Date of completion _____/______/________

1. How old are you (in full years)? _______
2. Which gender do you identify with? ( ) female ( ) male ( ) other
3. How would you define your *color/race*?

   ( ) white ( ) brown ( ) black ( ) Asian ( ) *Indigenous*
4. What is your marital status?
   ( ) Married ( ) *Cohabiting* ( ) Single ( ) *Divorced* ( ) Widowed
5. Do you have children? ( ) No ( ) Yes
6. What is your religion?
   ( ) none (go to question 8)
   ( ) Roman Catholic
   ( ) traditional Protestant
   ( ) Kardecist Spiritist
   ( ) Umbanda/Candomblé
   ( ) Eastern religions
   ( ) evangelical
   ( ) Jewish
   ( ) other
7. How important is religion in your life?
   ( ) very important ( ) important ( ) not very important ( ) unimportant
8. How long ago did you graduate (in full years)?
9. Do you have a medical residency or specialist title in *gynecology and obstetrics*?

   ( ) No ( ) Yes
10. In which region of Brazil do you currently provide medical care?
    ( ) North
    ( ) Northeast
    ( ) South
    ( ) Southeast
    ( ) *Central-West*
11. How long, in full years, have you been working at this hospital (include the length of your residency, if you have completed or are currently completing your

residency at this hospital)?

12. What is your main connection to this hospital?
    ( ) resident doctor
    ( ) *civil servant (statutory)*
    ( ) CLT employee
    ( ) cooperative contract
    ( ) temporary employment contract
    ( ) on-call doctor without employment contract
    ( ) self-employed
    ( ) other. Please specify:

13. What is your role at the hospital? (You may select more than one option)
    ( ) *day-shift physician*
    ( ) on-call
    ( ) service coordinator

14. Is this hospital public, private, or affiliated with the SUS?
    ( ) public
    ( ) private
    ( ) private affiliated with the SUS

15. Is this hospital located in a metropolitan area?
    ( ) Yes ( ) No ( ) I don't know

16. Is this hospital a teaching hospital (i.e., does it have undergraduate and/or specialization and/or residency students)?
    ( ) No ( ) Yes ( ) I don't know

17. On average, approximately how many vaginal deliveries and cesarean sections are performed at this hospital per month?
    ( ) Less than 50 per month
    ( ) 50 to 200 per month
    ( ) More than 200 per month
    ( ) I don't know

18. On average, approximately how many abortions are performed at this hospital per month?
    ( ) Less than 10 per month
    ( ) 10 to 50 per month
    ( ) More than 50 per month
    ( ) I don't know

19. Do you know what the cesarean section rate is at this hospital?
    ( ) Yes ( ) No (go to question 22)

20. What is the rate?
    ( ) Less than 20%
    ( ) 20 to 35%
    ( ) 36-60%
    ( ) More than 60%

21. Do you consider this rate to be appropriate?
    ( ) Yes, it is appropriate
    ( ) No, it is higher than it should be
    ( ) No, it is lower than it should be
    ( ) I don't know

22. Did you receive information about the following methods of labor induction during your medical training?

    a) Misoprostol
    ( ) no ( ) yes ( ) I don't remember

    b) Oxytocin
    ( ) no ( ) yes ( ) I don't remember

    c) Mechanical methods (*e.g., cervical balloon*)
    ( ) no ( ) yes ( ) I don't remember

23. Were you taught to perform *external cephalic version* for breech presentation fetuses during your medical training?
    ( ) No ( ) Yes ( ) I don't remember

24. Were you taught to assist in deliveries using forceps or vacuum extraction during your medical training?
    ( ) No ( ) Yes ( ) I don't remember

25. Were you taught to assist with deliveries in upright or vertical positions during your medical training?
    ( ) No ( ) Yes ( ) I don't remember

26. Were you taught about the use of misoprostol for abortion care during your medical training?
    ( ) No ( ) Yes ( ) I don't remember

27. Were you taught how to perform uterine curettage (D&C) during your medical training?
    ( ) No ( ) Yes ( ) I don't remember

28. Were you taught how to perform manual vacuum aspiration (MVA) during your medical training?
    ( ) No ( ) Yes ( ) I don't remember

29. Were you trained to provide assistance with the termination of pregnancy in cases permitted by law during your medical training?
    ( ) No ( ) Yes ( ) I don't remember

Now we will ask you some questions about training you have received at your workplace in the last 10 years. (*Do not include training received during your medical residency)*.

30. Have you ever received training on performing manual vacuum aspiration (MVA) at your workplace?
    ( ) No ( ) Yes ( ) I don't remember ( ) I am currently in medical residency
31. Have you ever received training to provide assistance with pregnancy termination in the legally permitted cases at your workplace?
    ( ) No ( ) Yes ( ) I don't remember ( ) I am currently in medical residency
32. Have you ever been trained in evidence-based best practices in labor and delivery care at your workplace?
    ( ) No ( ) Yes ( ) I don't remember ( ) I am currently in medical residency

---

There are technical documents issued by the Ministry of Health and the World Health Organization for obstetric care. Regarding these documents:

33. Are you familiar with the Ministry of Health's national guidelines for vaginal delivery care?
    ( ) I didn't know they existed
    ( ) I know they exist, but I have never read them
    ( ) I have read part of them
    ( ) I have read them in full
34. Are you familiar with the Ministry of Health's national guidelines on cesarean delivery?
    ( ) I didn't know they existed
    ( ) I know they exist, but I have never read them
    ( ) I have read part of them
    ( ) I have read them in full
35. Are you familiar with the 2018 WHO Intrapartum Care Guidelines for a Positive Childbirth Experience?
    ( ) I didn't know they existed
    ( ) I know they exist, but I have never read them
    ( ) I have read part of them
    ( ) I have read them in full
36. Are you familiar with the Ministry of Health's technical standards for humanized abortion care?
    ( ) I didn't know they existed
    ( ) I know they exist, but I have never read them
    ( ) I have read part of them
    ( ) I have read them in full

37. Are you familiar with the Ministry of Health's technical standards for prevention and treatment of harm from sexual violence against women and adolescents?
    ( ) I didn't know they existed
    ( ) I know they exist, but I have never read them
    ( ) I have read part of them
    ( ) I have read them in full

38. Are you familiar with the Ministry of Health's technical standards for care of women with anencephalic pregnancies?
    ( ) I didn't know they existed
    ( ) I know they exist, but I have never read them
    ( ) I have read part of them
    ( ) I have read them in full

Below are some statements about practices in labor and delivery care.
Please indicate whether you believe they are false, true, or if you do not know.

39. For low-risk women, the intake of fluids and light food during labor is a recommended practice.
    ( ) False ( ) True ( ) I don't know

40. The liberal or routine use of episiotomy is recommended for women undergoing spontaneous vaginal delivery.
    ( ) False ( ) True ( ) I don't know

41. A woman's preferred positions during labor, including upright or vertical positions, should be respected.
    ( ) False ( ) True ( ) I don't know

Below are some statements regarding the care of early fetal loss or legally permitted pregnancy termination.
Please indicate whether you believe they are false, true, or if you do not know.

42. The ethical guidelines governing professional practice define conscientious objection as a right that allows healthcare professionals to refuse to assist in legally permitted pregnancy termination under any circumstance, even if no other professional is available to ensure the patient receives care.
    This statement is:
    ( ) False ( ) True ( ) I don't know

43. Regarding the use of misoprostol for managing early fetal loss or pregnancy termination in legally permitted cases:

a. The greater the gestational age, the higher the dose that should be used.
( ) False ( ) True ( ) I don't know

b. Vaginal administration causes fewer side effects than oral or sublingual routes.
( ) False ( ) True ( ) I don't know

c. In first-trimester abortion induction, the best outcomes are achieved with 400 mcg orally and 400 mcg vaginally per administration.
( ) False ( ) True ( ) I don't know

d. Misoprostol can be used without hospitalization for pregnancies of up to nine weeks.
( ) False ( ) True ( ) I don't know

---

44. Regarding manual vacuum aspiration (MVA) in the care of early fetal loss or legally permitted pregnancy termination:

a) MVA is the preferred method for uterine evacuation up to 12 weeks of gestation.
( ) False ( ) True ( ) I don't know

b) MVA has higher complication rates than traditional curettage.
( ) False ( ) True ( ) I don't know

c) MVA is the method of choice in cases of infected first-trimester abortion.
( ) False ( ) True ( ) I don't know

We would like your opinion on current Brazilian legislation regarding obstetric care:

45. Under current Brazilian law, nurses and midwives are qualified to assist in uncomplicated vaginal deliveries for low-risk pregnancies. Do you agree with this law?
( ) I agree ( ) I disagree ( ) I have no opinion

46. According to a resolution by the Federal Council of Medicine, elective cesarean sections should not be performed before 39 weeks of gestation. Do you agree with this recommendation?
( ) I agree ( ) I disagree ( ) I have no opinion

47. Under current Brazilian law, pregnancy termination is not considered a crime in the following cases:

- pregnancy resulting from rape
- pregnancy posing a risk to the woman's life
- fetal anencephaly

Do you agree with this legislation?

47.a. In cases of pregnancy resulting from rape?
( ) I agree ( ) I disagree ( ) I have no opinion

47.b. In cases where the pregnancy poses a risk to the woman's life?
( ) I agree ( ) I disagree ( ) I have no opinion

47.c. In cases of fetal anencephaly?
( ) I agree ( ) I disagree ( ) I have no opinion

---

48. In your opinion, should pregnancy termination be allowed in the following situations?
(Please indicate whether you agree, disagree, or have no opinion for each)

a) The fetus has a severe congenital malformation incompatible with life outside the womb.
( ) I agree ( ) I disagree ( ) I have no opinion

b) The contraceptive method used has failed.
( ) I agree ( ) I disagree ( ) I have no opinion

c) The woman does not have the financial means to raise the child.
( ) I agree ( ) I disagree ( ) I have no opinion

d) The pregnancy severely impacts the woman's physical and/or mental health.
( ) I agree ( ) I disagree ( ) I have no opinion

e) The woman does not want to interrupt her studies or career.
( ) I agree ( ) I disagree ( ) I have no opinion

f) In any circumstance, if the woman decides so.
( ) I agree ( ) I disagree ( ) I have no opinion

We would like your opinion on some statements related to labor and delivery care:

49. The natural progression of labor without interventions unnecessarily prolongs the process and causes excessive exhaustion for the mother.
( ) I agree ( ) I disagree ( ) I have no opinion

50. In your opinion, for a woman with a low-risk pregnancy, what is the safest type of delivery for both mother and baby, considering short-, medium-, and long-term outcomes?
( ) Vaginal delivery is safer
( ) Cesarean section is safer
( ) Both are equally safe
( ) I have no opinion

51. A pregnant woman should have the right to request a cesarean section even without medical indication.
( ) I agree ( ) I disagree ( ) I have no opinion

52. If an adverse event or complication occurs, do you think it is more likely that the physician will be sued after attending a:
( ) Vaginal delivery
( ) Cesarean section
( ) It does not depend on the mode of delivery
( ) I have no opinion

53. What do you believe are the main factors contributing to Brazil's high cesarean rate? (You may select more than one)
( ) Fear of lawsuits due to poor outcomes in vaginal deliveries
( ) Cesarean section upon maternal request
( ) Obstetrician convenience
( ) Perceived greater safety of cesarean surgery
( ) Low reimbursement for vaginal delivery
( ) Urban violence (e.g., fear of attending deliveries at night)
( ) Unnecessary cesarean indications
( ) Lack of infrastructure or adequate multidisciplinary teams
( ) Other. Which? ___________

54. In your experience, what are the main reasons women request cesarean sections? (You may select more than one)
( ) Fear of pain
( ) Fear of vaginal delivery
( ) Convenience/scheduling flexibility
( ) To undergo tubal ligation
( ) To be attended by their chosen doctor
( ) To preserve the perineum
( ) Perceived increased safety for the woman
( ) Perceived increased safety for the baby
( ) To ensure a maternity bed
( ) Previous negative experience with vaginal delivery
( ) Previous positive experience with cesarean delivery
( ) Other. Which? ___________

55. Have you ever performed a cesarean section out of fear that a complication in a vaginal delivery could lead to a lawsuit?
( ) Yes ( ) No ( ) I prefer not to say

56. Have you ever opted for a cesarean instead of ***an instrument-assisted vaginal delivery*** (e.g., vacuum or forceps), when indicated, out of concern for legal consequences?
( ) Yes
( ) No
( ) I never perform instrument-assisted vaginal deliveries

Now moving to questions about early fetal loss and legal pregnancy termination:

57. Please indicate your opinion on the advantages and disadvantages of medical abortion compared to surgical abortion. For each statement below, indicate if it's an advantage, disadvantage, neither, or if you have no opinion.

a) Medical abortion avoids the use of anesthesia
( ) Advantage ( ) Disadvantage ( ) Neither ( ) No opinion

b) Medical abortion can be done at home
( ) Advantage ( ) Disadvantage ( ) Neither ( ) No opinion

c) Medical abortion takes more time to complete
( ) Advantage ( ) Disadvantage ( ) Neither ( ) No opinion

58. Please indicate your opinion on the advantages and disadvantages of MVA compared to curettage. For each item below, mark the appropriate option:

a) MVA requires a shorter hospital stay
( ) Advantage ( ) Disadvantage ( ) Neither ( ) No opinion

b) MVA less often requires cervical dilation
( ) Advantage ( ) Disadvantage ( ) Neither ( ) No opinion

c) MVA requires less pain management
( ) Advantage ( ) Disadvantage ( ) Neither ( ) No opinion

59. In your opinion, if a woman is known to have induced an abortion, how should a healthcare professional proceed?
( ) Maintain professional confidentiality
( ) Report the crime to authorities
( ) Depending on the situation, may or may not report
( ) I have no opinion

60. In your opinion, should doctors have the right to invoke conscientious objection to refuse assistance in legal pregnancy terminations?
( ) Yes, under any circumstance
( ) Yes, but only if another provider is available to ensure care
( ) No
( ) I have no opinion

Now we will ask you some questions about your professional practice:

61. At your workplace, is there a protocol for humanized, evidence-based care for women in labor?
    ( ) Yes
    ( ) No (go to question 63)
    ( ) I don't know (go to question 63)

62. Do you believe this protocol is used as a reference by you and your colleagues?
    ( ) Yes, always
    ( ) Yes, sometimes
    ( ) Never
    ( ) I don't know

63. Are obstetric nurses or midwives available to assist in the care of low-risk labor and deliveries at your workplace?
    ( ) Yes, always
    ( ) Yes, sometimes
    ( ) No (go to question 65)
    ( ) I don't know (go to question 65)

64. In cases of low-risk pregnancies with an obstetric nurse/midwife available to attend the birth, you:
    ( ) Prefer to provide the care because you consider it a medical responsibility
    ( ) Do not provide direct care, but make a point to be present to ensure everything is done correctly
    ( ) Do not provide direct care, but remain available in case of complications

---

65. Indicate your usual practices regarding labor and delivery care:

a) Encourage free ambulation and mobility during labor
( ) Always ( ) Sometimes ( ) Never

b) Encourage intake of clear liquids or light diet during labor
( ) Always ( ) Sometimes ( ) Never

c) Encourage non-pharmacological pain relief methods (e.g., shower, bath, massage, birth ball, etc.)
( ) Always ( ) Sometimes ( ) Never

d) Encourage the presence of a companion chosen by the woman during labor
( ) Always ( ) Sometimes ( ) Never

e) Perform early amniotomy (before 4 cm dilation)
( ) Always ( ) Sometimes ( ) Never

f) Encourage upright positions during delivery
( ) Always ( ) Sometimes ( ) Never

g) Perform episiotomy
( ) Always ( ) Sometimes ( ) Never

h) Promote **immediate and uninterrupted skin-to-skin contact** between mother and baby
( ) Yes, for at least 1 hour
( ) Yes, for less than 1 hour
( ) Never

---

66. Does your workplace have a protocol for managing abortion cases?
    ( ) Yes
    ( ) No (go to question 68)
    ( ) I don't know (go to question 68)

67. Do you believe this protocol is used as a reference by you and your colleagues?
    ( ) Yes, always ( ) Yes, sometimes ( ) Never ( ) I don't know

68. Is manual vacuum aspiration (MVA) available at your workplace?
    ( ) Yes, always
    ( ) Yes, sometimes
    ( ) No (go to question 70)
    ( ) I don't know (go to question 70)

69. When MVA is indicated, do you use it in your workplace?
    ( ) Yes, whenever available (go to question 70)
    ( ) No

69.a. Why not? (Select all that apply)
( ) I feel safer performing curettage than MVA
( ) I have never been trained to perform MVA
( ) I believe curettage is more effective
( ) Other. Please specify: __________

---

70. If MVA is not available at your workplace, is electric vacuum aspiration performed?
    ( ) Yes ( ) No ( ) I don't know

71. Have you ever participated in assisting a woman with legally permitted pregnancy termination, either in this hospital or elsewhere?
    ( ) Yes ( ) No

72. Does this facility provide care to women seeking termination of pregnancy in legally authorized cases?
    ( ) Yes ( ) No (go to question 77)
    ( ) I don't know (go to question 77)

73. Are you part of the care team that assists in legally authorized terminations?
    ( ) Yes, in all situations (go to question 74)
    ( ) Only in some situations (go to question 73a)
    ( ) Never (go to question 73b)
    ( ) I prefer not to say (go to question 78)

73a. Which situations? (You may select more than one)
( ) Rape
( ) Risk to the woman's life
( ) Anencephaly
(Proceed to question 74)

73b. Reason for not participating (Select all that apply):
( ) It goes against my religious beliefs
( ) It causes me emotional distress
( ) I fear being discriminated against
( ) I invoke conscientious objection
( ) Other. Please specify: __________
(Proceed to question 78)

---

74. At this facility, is a police report or court order required in order to provide legal termination of pregnancy (in cases of rape, anencephaly, or risk to life)?
    ( ) No
    ( ) Only police report
    ( ) Only court order
    ( ) Both
    ( ) I don't know

75. Does this facility hold multidisciplinary team meetings to discuss care for hospitalized women undergoing legally permitted terminations?
    ( ) Always
    ( ) Sometimes
    ( ) Never
    ( ) I don't know

76. Do professionals at your hospital claim conscientious objection in order to avoid participating in care for legal terminations?
    ( ) Yes, most professionals
    ( ) Yes, some professionals
    ( ) No
    ( ) I don't know
    (Proceed to question 78)

77. If this hospital were to begin offering services for legal pregnancy terminations, would you agree to join the care team?
( ) Yes, in all cases (go to question 78)
( ) Only in some cases (go to question 77a)
( ) Never (go to question 77b)
( ) I prefer not to say (go to question 78)

77a. In which situations would you participate? (Select all that apply)
( ) Rape
( ) Risk to the woman's life
( ) Anencephaly
(Proceed to question 78)

77b. Why would you not participate? (Select all that apply)
( ) It goes against my religious beliefs
( ) It causes me emotional distress
( ) I fear being discriminated against
( ) I would claim conscientious objection
( ) Other. Please specify: __________

---

78. In your opinion, do women who have abortions at this maternity hospital experience discrimination from healthcare professionals?
( ) Yes, from most professionals
( ) Yes, from some professionals
( ) No
( ) I don't know

79. During your career, have you ever assisted a woman who had an induced or suspected induced abortion?
( ) Yes
( ) No (go to question 82)
( ) I prefer not to say (go to question 82)

80. What feelings did you have toward the woman?
( ) Empathy
( ) Reproach, because she committed an illegal act
( ) Indifference, it’s her personal issue
( ) I had mixed feelings
( ) I don’t remember

81. In your practice, when you discover that a woman has had an abortion, how do you proceed?
( ) I maintain professional confidentiality
( ) I report the crime to the authorities
( ) Depending on the case, I may or may not report it
( ) I have no opinion

82. In your opinion, what are the main enablers of your work as an obstetrician in this maternity hospital? (Select all that apply)
( ) Commitment of hospital management
( ) Commitment of professionals
( ) Shared governance strategies (e.g., collective discussion and decision-making spaces)
( ) Positive hospital environment
( ) Availability of supplies
( ) Available equipment and furniture
( ) Availability of a multidisciplinary team
( ) Adequate staffing levels
( ) Implementation of clinical protocols
( ) Patient volume
( ) Other. Please specify: __________

83. In your opinion, what are the main barriers or challenges to your work as an obstetrician in this maternity hospital? (Select all that apply)
( ) Commitment of hospital management
( ) Commitment of professionals
( ) Shared governance strategies
( ) Physical environment of the hospital
( ) Supply shortages
( ) Lack of equipment or furniture
( ) Lack of multidisciplinary support
( ) Inadequate number of professionals
( ) Lack of established protocols
( ) High patient volume
( ) Other. Please specify: __________

Please use the space below to share any additional comments or observations.

Thank you very much for your cooperation!
